# Genome Sequence of *Acinetobacter baumannii* Strain 10441_14 Belonging to ST451, Isolated from India

**DOI:** 10.1128/genomeA.01322-15

**Published:** 2015-11-05

**Authors:** Sunil Kumar, Prashant P. Patil, Samriti Midha, Pallab Ray, Prabhu B. Patil, Vikas Gautam

**Affiliations:** a Department of Medical Microbiology, Post Graduate Institute of Medical Education and Research, Chandigarh, India; b Laboratory of Bacterial Genomics and Evolution, CSIR-Institute of Microbial Technology, Chandigarh, India

## Abstract

*Acinetobacter baumannii* resistance to carbapenems is of global concern. Here, we report the 3.9 Mb draft genome of a cerebrospinal fluid isolate of *A. baumannii* strain 10441_14 which is carbapenem resistant and belongs to ST451. This genome will further help in the understanding of the drug resistance mechanism, epidemiology, and pathology of this bacterium.

## GENOME ANNOUNCEMENT

*Acinetobacter baumannii* is one of the leading causes of nosocomial infections worldwide (1, 2). Multidrug resistance is a major public health concern, and is even more so in *A. baumannii* as it has the ability to develop resistance against almost all available antimicrobials (3). Carbapenems are widely being used as a treatment option for infections of *A. baumannii*. However, carbapenem-resistant *A. baumannii* has emerged as a serious challenge to health care systems globally as few treatment options are available (4).

Here, we present the genome sequence of the *A. baumannii* strain 10441_14, isolated from the cerebrospinal fluid of patient admitted into the neurosurgery ward of the Post Graduate Institute of Medical Education and Research (PGIMER), Chandigarh, a tertiary care hospital in northern part of India. Multilocus sequence typing by scheme defined by Bartual et al. (5) shows that isolate 10441_14 belongs to ST451. ST451 belongs to clonal complex 92 (CC92), which corresponds to the international clone lineage II that is commonly observed among carbapenem-resistant *A. baumannii* in hospitals worldwide (6, 7).

For whole-genome sequencing, genomic DNA was isolated using a ZR Fungal*/*Bacterial DNA miniprep Kit (Zymo Research). The quality of DNA was assessed using NanoDrop (Thermo Scientific, Wilmington, MA, USA) and agarose gel electrophoresis and concentration were estimated using a Qubit 2.0 Fluorometer (Life Technologies). An Illumina sequencing library of genomic DNA was prepared using a Nextera XT sample preparation kit (Illumina, Inc., San Diego, CA, USA) with dual indexing adapters. The Illumina sequencing library was sequenced on an in-house Illumina Miseq (Illumina, Inc., San Diego, CA, USA) platform using the 2 × 250 bp paired-end configuration. Sequencing of the DNA generated 2,521,072 reads, accounting for 365,553,621 bp. Raw reads were assembled using CLC Genomics Workbench v7.5 (CLC bio, Aarhus, Denmark) into 136 contigs with a genome size of 3,909,844 bp and average coverage 92.78×. *N*_50_ for the assembly is 61,813 bp and the genome has an average G+C content of 38.93%. The genome was annotated using the NCBI Prokaryotic Genome Annotation Pipeline (http://www.ncbi.nlm.nih.gov/genome/annotation_prok/), which predicted 3,775 genes, 63 tRNAs, and 3 rRNAs.

A complete 16s rRNA gene sequence was retrieved from the genome by using RNAmmer v1.2 (8) and the average nucleotide identity (ANI) was calculated using Jspecies v1.2.1 (9). The *A. baumannii* strain 10441_14 has 16s rRNA identities of 99.97% and 97.7% ANI with the type strain *A. baumannii* ATCC 19606^T^.

The genome information of *A. baumannii* 10441_14 will accelerate our understanding of the whole-genome-based phylogenetic and the comparative analysis of this globally important nosocomial pathogen.

### Nucleotide sequence accession numbers.

The draft genome sequence of *Acinetobacter baumannii* 10441_14 is available in DDBJ/EMBL/GenBank under accession no. LGYW00000000. The version described in this paper is the first version, LGYW01000000.
